# MIRKB: a myocardial infarction risk knowledge base

**DOI:** 10.1093/database/baz125

**Published:** 2019-11-04

**Authors:** Chaoying Zhan, Manhong Shi, Rongrong Wu, Hongxin He, Xingyun Liu, Bairong Shen

**Affiliations:** 1 Centre for Systems Biology, Soochow University, Suzhou 215006, China; 2 College of Information and Network Engineering, Anhui Science and Technology University, Fengyang, Anhui 233100, China; 3 Institutes for Systems Genetics, West China Hospital, Sichuan University, Chengdu 610041, China

## Abstract

Myocardial infarction (MI) is a common cardiovascular disease and a leading cause of death worldwide. The etiology of MI is complicated and not completely understood. Many risk factors are reported important for the development of MI, including lifestyle factors, environmental factors, psychosocial factors, genetic factors, etc. Identifying individuals with an increased risk of MI is urgent and a major challenge for improving prevention. The MI risk knowledge base (MIRKB) is developed for facilitating MI research and prevention. The goal of MIRKB is to collect risk factors and models related to MI to increase the efficiency of systems biological level understanding of the disease. MIRKB contains 8436 entries collected from 4366 articles in PubMed before 5 July 2019 with 7902 entries for 1847 single factors, 195 entries for 157 combined factors and 339 entries for 174 risk models. The single factors are classified into the following five categories based on their characteristics: molecular factor (2356 entries, 649 factors), imaging (821 entries, 252 factors), physiological factor (1566 entries, 219 factors), clinical factor (2523 entries, 561 factors), environmental factor (46 entries, 26 factors), lifestyle factor (306 entries, 65 factors) and psychosocial factor (284 entries, 75 factors). MIRKB will be helpful to the future systems level unraveling of the complex mechanism of MI genesis and progression.

## Introduction

Myocardial infarction (MI) is defined as myocardial necrosis due to coronary ischemia. MI is a common cardiovascular disease and a leading cause of death worldwide. In the USA, someone will develop MI approximately every 40 seconds. It has been estimated that 605 000 Americans will have a new acute MI and another 200 000 will have a recurrent MI event each year (1). Many advanced approaches have been developed for the management of patients with MI, such as thrombolytic therapy and interventional therapy (2–4). However, MI remains a major problem worldwide. The main risk factors for MI include genetic susceptibility and non-genetic factors such as hypertension, obesity, diabetes and lifestyle (5–10). Many studies have shown that smoking is not only a risk factor for the onset of MI but also a predictor of a poor prognosis for MI (7, 11, 12). Some other studies demonstrated that smoking is not significantly associated with the onset and prognosis of MI (13–16). It was reported that high physical activity (PA) is a protective factor for 1-year readmission due to non-cardiovascular disease in patients with MI (17). PA is also a protective factor for the incidence of MI (18), although another study indicated that PA is not significantly associated with the risk of acute MI (19). Family history of premature coronary artery disease plays an important role in the development of MI (10). Many studies showed that identifying individuals with an increased risk of MI is a major challenge for enhancing prevention.

With our increased understanding of the pathogenesis of MI, the applications of biomarkers for early diagnosis, treatment strategies and prognosis evaluation in MI have become very popular in recent researches. Biomarkers are classified as molecular markers, imaging markers and physiological markers according to their properties, and they are also classified as diagnostic markers, therapeutic markers and prognostic markers according to their clinical applications. Clinically, cardiac troponin and creatine kinase-MB can be used as biomarkers for the diagnosis, treatment and prognosis of MI (20–24). The fragmented QRS complex in the electrocardiogram (ECG) is a prognostic marker for in-hospital life-threatening arrhythmic complications in ST-segment elevation MI (STEMI) patients (25). In recent years, microRNAs have been employed not only as biomarkers of MI development, but also as predictors for treatment monitoring and poor prognosis (26, 27). It is well known that microRNA-1 (miR-1) plays an important role in heart disease, and studies have shown that miR-1 may be employed as a biomarker for the diagnosis of acute MI (28, 29). In addition, miR-133 has been used as a marker for the diagnosis of acute non-STEMI (30), and Cortez-Dias *et al.* showed that the miR-122-5p/miR-133b ratio is a predictor of major adverse cardiac events in patients with acute STEMI (31).

Based our knowledge, no specific database containing risk factors and biomarkers related to MI is available, we therefore constructed here a knowledge base i.e. the MI risk knowledge base (MIRKB) for the understanding and prediction of the MI risk. The goal of MIRKB was to collect risk factors and models related to MI to improve the systems biological level understanding of MI. MIRKB is manually curated and constantly updated by the authors in order to include new data as soon as they are available. MIRKB also allows users to contribute to the project through an online data submission form.

## Database description

### Data collection and statistics

Regarding data collection, all the data for our MIRKB were collected from the public database PubMed (www.ncbi.nlm.nih.gov/pubmed) by human text mining. We conducted the search of PubMed using keywords such as ‘MI‘ and ‘biomarker or marker or indicator or predictor or risk factor or risk model‘, etc.

We set the following criteria for the studies included in our database: (i) epidemiological population studies of MI; (ii) studies associated with the risk, diagnosis, prognosis and treatment of MI; (iii) studies that used one or more statistics to evaluate relationships with MI (e.g. sensitivity, specificity, positive predictive value, negative predictive value, area under the curve of the receiver operating characteristic curve, *P*-value, hazard ratio, odds ratio and risk ratio and their 95% confidence intervals, etc.). Duplicate studies, animal studies, reviews, case reports, letters, studies without full texts, and studies with defective designs and poor quality were then excluded. In addition, we classified the MI patients into young and elderly populations according to the descriptions reported in the researches and the patients younger than 65 years old were classified as young population and the others were grouped to elderly population.

**Figure 1:**
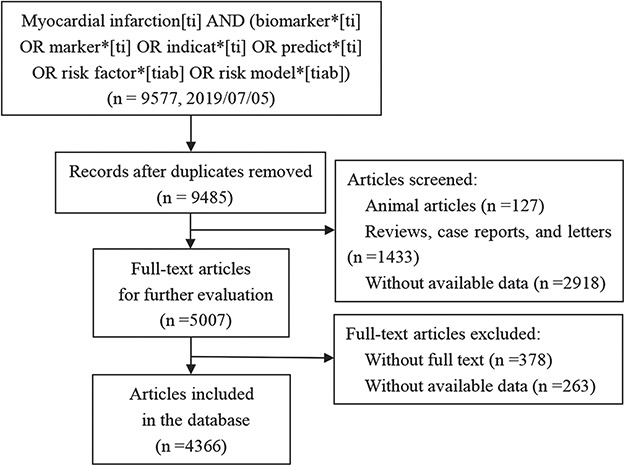
The flowchart for the manual collection of MI risk factors and models.

Based on these criteria, 9577 articles from PubMed have been collected as the original data for our database MIRKB before 5 July 2019. There were still 9485 articles remained after removing the duplicates. Since only the literature related to the risk, diagnosis, prognosis and treatment of MI in human was considered to be collected into our database, we excluded animal articles (*n* = 127), reviews, case reports and letters (*n* = 1433), articles without available data (*n* = 2918), after which 5007 original articles remained. By reading the full text of articles, there were 4366 original articles finally after we removed articles without full text (*n* = 378) and articles without available data (*n* = 263). The detail about the literature collection is shown as a flowchart in Figure 1. The next step was to extract information from the 4366 articles. This step was manually curated to ensure that the associations between risk factors, risk models and MI existed and were significant and that sufficient details were recorded for the associations. If some studies prove that certain risk factors are not significantly associated with MI, other studies have clearly indicated that they have statistical relationships, and these studies remain. Finally, we integrated the collected data, and when a risk factor has multiple names, it is labeled with a uniform official name. For example, brain natriuretic peptide (BNP), also known as B-type natriuretic peptide, was named by using ‘BNP’ according to NCBI protein.

The MIRKB database contains a total of 8436 entries collected from 4366 articles in PubMed with 7902 entries for 1847 single factors, 195 entries for 157 combined factors and 339 entries for 174 risk models. The single factors are classified into the following five categories based on their characteristics: molecular factor (2356 entries, 649 factors), imaging (821 entries, 252 factors), physiological factor (1566 entries, 219 factors), clinical factor (2523 entries, 561 factors), environmental factor (46 entries, 26 factors), lifestyle factor (306 entries, 65 factors) and psychosocial factor (284 entries, 75 factors). There were 572 entries for young population and 217 entries for elderly population based on the research population. According to the applications involved, there were 1755 entries for risk assessment, 279 entries for diagnosis, 6165 entries for prognosis, 112 entries for treatment and 127 entries for others. Descriptive statistics for the database are shown in Table 1.

**Table 1:** Statistics for the important fields included in MIRKB

Data content	Number of entries	Number of categories	Data content	Number of entries
**Classification**			**Population**	
Single factor	7902	1847	Young	572
Molecular factor	2356	649	Elderly	217
Protein	1274	277	**Application**	
DNA	423	162	Risk assessment	1755
RNA	82	56	Diagnosis	279
Other	577	154	Prognosis	6163
Imaging	821	252	Treatment	112
Physiological factor	1566	219	Other	127
Clinical factor	2523	561	**MI type**	
Disease history	1780	370	Disease phase	
Family history	39	5	Acute	6282
Treatment history	520	134	Old	154
Other	184	52	Lesion range
Environmental factor	46	26	Transmural	30
Lifestyle factor	306	65	Subendocardial	0
Behavioral hobby	163	10	Infarction location	
Eating habit	104	46	Anterior	326
Exercise habit	34	6	Inferior	73
Routine	5	3	Other sites	0
Psychosocial factor	284	75	ECG expression
Combined factors	195	157	ST-segment elevation	2859
Risk model	339	174	Non-ST-segment elevation	195
			**Clinical type**	
			Type I	8
			Type II	6
			Type III	0
			Type IV	27
			Type V	18

**Figure 2:**
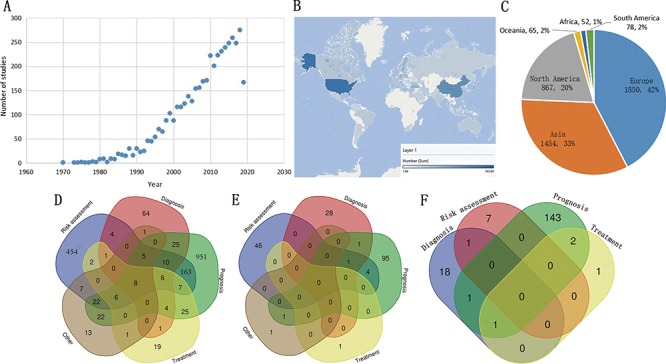
**Examples of statistical analyses from the MIRKB.** (**A**) Study number distribution according to year of publication; (**B** and **C**) study number distribution according to research region; (**D–F**) showed number distribution of single factors, combined factors and risk models according to their application, respectively.

By analyzing the entries in the MIRKB, it indicates that the related works keep increasing year by year (Figure 2a), suggesting that research on MI remains one of the hottest topics in the field of complex cardiovascular diseases all the time. Following this tendency, it is foreseeable that more studies related to MI will be published for the risk assessment, diagnosis, prognosis and treatment of MI, and we will integrate them into MIRKB. The MIRKB has collected the studies related to MI from 83 countries in 6 continents. The country with the largest number of studies is USA, followed by China and others (Figure 2b). The continent with the largest number of studies is Europe, followed by Asia and North America (Figure 2c). The risk factors and models are mostly for prognosis in terms of their clinical application distribution, followed by risk assessment and diagnosis (Figure 2d–f).

### Database architecture

**Figure 3:**
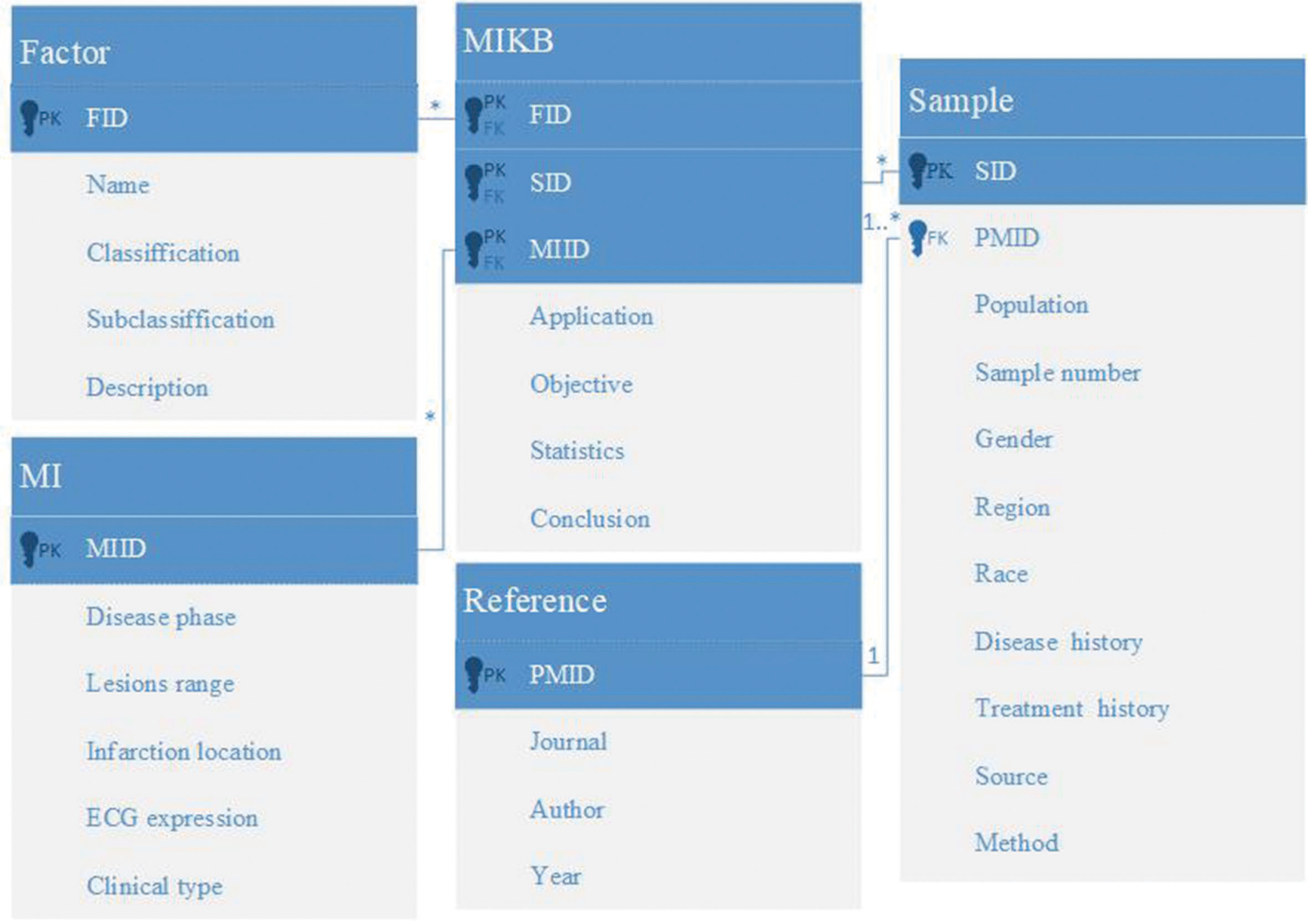
Entity relationship diagram of MIRKB.

MIRKB is a relational database, and it includes information regarding risk factors, models, MI, references, samples and the relationships between them. The entity relationships of the MIRKA are depicted in Figure 3. The conceptual architecture described was designed to facilitate the inclusion of new factors related to MI when updating the database.

### Database implementation details

The MIRKB applied PHP (http://www.php.net/) development technology with MySQL (http://www.mysql.com) as the background database running on an Apache server, where HTML and CSS scripting languages were employed for client side coding, and the Web platform is based on the Browser/Server (B/S) mode.

### Web framework

**Figure 4:**
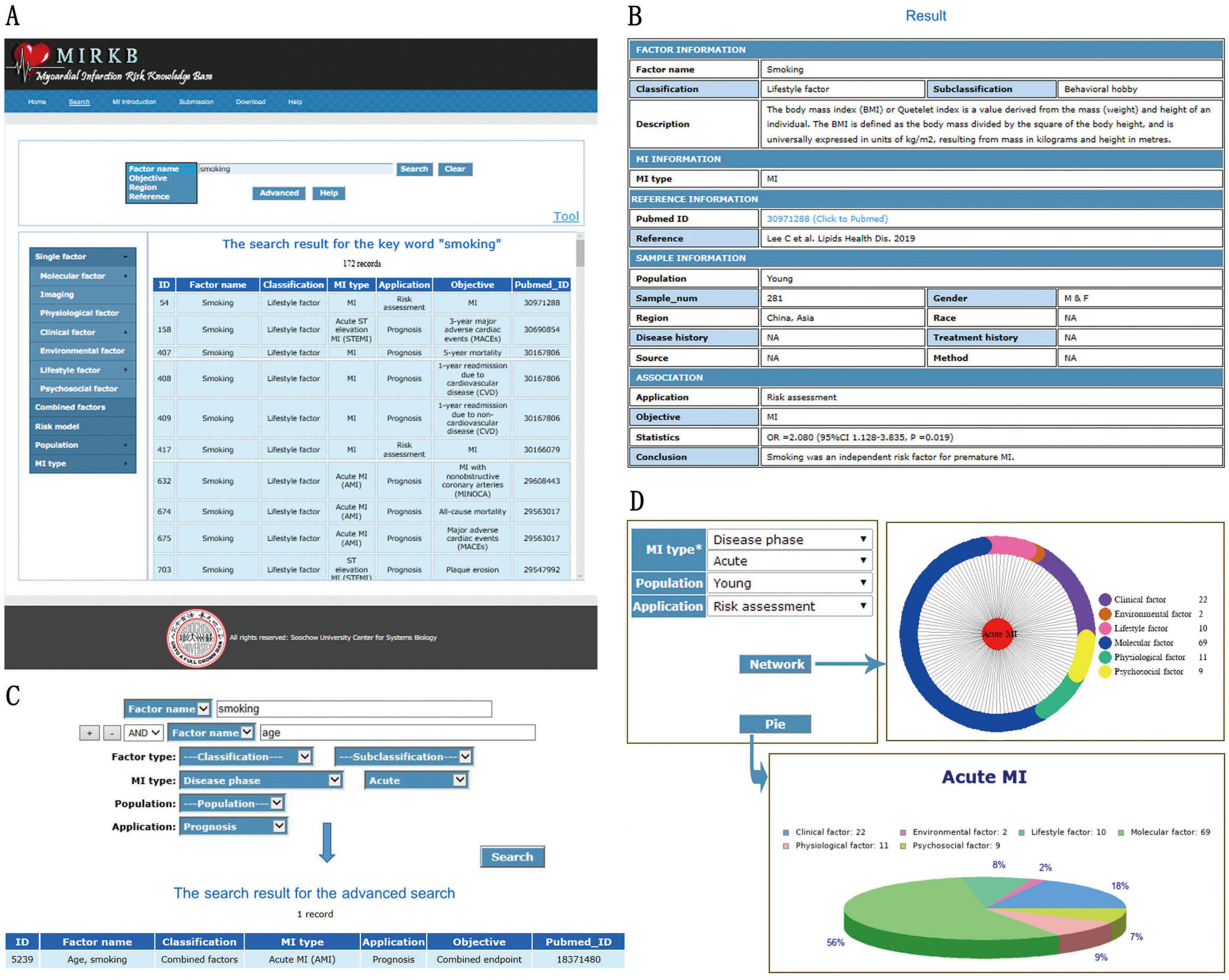
**MIRKB interface.** (**A**) ‘Search’ page. (**B**) ‘Detailed search results’ interface. (**C**) Example of ‘Advanced search’. (**D**) ‘MI introduction’ page.

The web framework of the MIRKB has six components: (i) the ‘Home’ page for providing brief introduction of MIRKB, (ii) the ‘Search’ page provides navigation bar search, keywords search, and advanced search for browsing and retrieving data, (iii) the ‘MI introduction’ page provides the definition of different MI types and external links (Wikipedia and PubMed website), (iv) the ’Submission’ page for users to submit new data related to MI, (v) the ‘Download’ page for users to download all the data of MIRKB and (vi) the ‘Help’ page for users to make full use of MIRKB (Figure 4a).

### Navigation bar and keywords search

The ‘Search’ page comprises a search box, navigation bar and results list (Figure 4a). A simple text search and a navigation bar are provided for fast data retrieval based on a single keyword. The database can be searched using the risk factor name, objective, region and reference (including the first author’s name, published journal and year of research), and it may be browsed by risk factor classification, research population and MI type. The screenshot in Figure 4a shows an example of a keyword search, where we searched records related to smoking and 172 entries were retrieved. Clicking on the results list allowed us to enter the ‘Detailed search results’ interface (Figure 4b). The results table includes data such as risk factor information (name, classification, subclassification and description), MI type, information about published references (PubMed ID, first author’s name, journal and year of research), sample information (population, sample number, sample gender, region, race, disease history, treatment history, source and method), application, objective, statistics and conclusion of the research. The table also provides PubMed link of the related studies.

### Advanced search

The ‘Advanced’ button can be clicked to make the ‘Advanced search’ (Figure 4a). The advanced search is a more powerful method for querying a database. Users can combine and specify different search terms to obtain more accurate results. The screenshot in Figure 4c shows an example of the advanced search, where we searched details of the relationship between the combination of smoking and age, and the prognosis of acute MI, and one record was retrieved.

### Statistical tool

MIRKB also provides a web tool for data statistics. If you want to use this tool, you need to click ‘Tool’ link(Figure 4a), and then the web page will jump into the ‘Tool’ page (Figure 4d). First and foremost, you need to choose a type of MI. And then, choose the research population and the application of the risk factors which you want to know. Finally, choose one of the buttons to draw the chart you want. This tool provides two charts including a network diagram and a pie chart. In this example, based on these two graphs, we can see that among the young population, molecular factors are the most in the risk factors related to the risk assessment of acute MI.

### MI introduction page

The ‘MI introduction’ page has a navigation bar and an information box. A detailed introduction to MI can be accessed by clicking on the left navigation bar to enter Wikipedia, including details of the terminology, signs and symptoms, causes, mechanism and diagnosis of MI. MI has different classification methods according to the disease phase, lesion range, infarction location, ECG expression and clinical type. According to the disease phase, MI can be divided into acute and old MI. MI can be categorized as transmural and subendocardial MI based on the lesion range of the infarction. According to the ECG expression, MI can be differentiated into silent, ST-segment elevation, non-ST-segment elevation, Q wave and non-Q wave MI. MI can be classified as anterior, inferior and MI of other sites according to the location. MI can be classified as type I, type II, type III, type IV (type IV includes type IVa and IVb) and type V MI according to the clinical type. The information box on the right-hand side provides access to the detail of the MI types.

**Table 2:** Comparisons of other biomedical databases

	CBD	AGD	GIDB	MIRKB
Purpose of the database	A database for collecting biomarkers related to the diagnosis, treatment or prognosis of colorectal cancer from the literature	A database for collecting genes associated with aneurysm in human, rat and mouse from both the literature and data available in public databases	A database for collecting genes associated with gastrointestinal cancer from both the literature and data available in public databases	A database for collecting risk factors and risk models related to the diagnosis, treatment or prognosis of MI from literature
Data resource	Scientific literature	Scientific literature and other scientific resources	Scientific literature and other scientific resources	Scientific literature
Data collection	Manually curated	Manually curated	Automated text mining	Manually curated
Analysis function	No	No	Yes	Yes

### Submission page

Another important feature implemented in MIRKB allows collaborative extension of the knowledge base. Currently, MIRKB contains relevant information from PubMed before 5 July 2019. However, MIRKB is not real-time updated and related data may not be updated in time, thus we implemented a submission function to allow users to help us to improve the MIRKB. Users can contribute to the project by submitting new MI data, which can be uploaded via the online submission form and added to the database after careful review.

### Download page

The ‘Download’ page lists all the data in MIRKB that is available for downloading.

### Comparisons with other databases

We compared our MIRKB with other related biomedical databases, such as colorectal cancer (CRC) biomarker database (CBD) (32), aneurysm gene database (AGD) (33) and gastrointestinal (GI) cancer knowledge database (GIDB) (34) listed in Table 2. The advantages of MIRKB are as follows: (i) the use of manual text-mining to make data in MIRKB more accurate, compared with automatic text-mining based databases, (ii) provide a statistical tool for the analysis between MI and risk factors and (iii) the data collection range is wide, including molecular factors, imaging, physiological factors, clinical factors, environmental factors, lifestyle factors and psychosocial factors, and combine factors as well as risk models. The MIRKB also provides users with very friendly interfaces and interactive tools, by which users can get the information they are interested in by browsing, keyword search or advanced search. At present, MIRKB only includes data extracted from PubMed, not EMBASE, EBSCO, Web of Science, OVID, etc., which may result in some valuable data not being entered into the database. Taken together, the MIRKB is an integrated research platform for studying the interactions of factors, risk models and MI, which is a unique and will be helpful to the future modeling and understanding of MI.

## Conclusion and discussion

MI is a common disease that poses a serious threat to human health. After an MI, lots of cardiomyocytes are necrotic, gradually replaced by fibrous tissue, resulting left ventricular remodeling, which eventually leads to congestive heart failure (35). With the rapid development of modern medicine, more and more methods are available for the treatment of MI, such as coronary intervention, bypass surgery, drugs, etc. Myocardial necrosis is an irreversible process and these treatments can prevent the procession of remodeling to a certain extent, but cannot repair or reverse the necrotic myocardium, let alone promote myocardial regeneration (36). Therefore, the early prevention, detection, diagnosis and treatment of MI are imperative. A growing number of risk factors have been shown to be associated with the development, progression, treatment and prognosis of MI, and different types of MI could have different risk factors. It is still a challenge to identify the precision risk factors for different types of MI for the early diagnosis, treatment and prognosis of the disease. With the accumulation of MI studies the systems biological level understanding of MI is becoming reality also necessary. The deep phenotyping based on combination of trans-omics factors will facilitate the precision and personalized diagnosis, prognosis and treatment of MI.

We created MIRKB to provide more comprehensive and accurate information to facilitate MI research at systems level. With MIRKB, researchers may obtain specific knowledge for risk factors, protective factors or biomarkers of typical MI types. Users can search for combined factors or risk models to predict the diagnosis, prognosis or treatment of MI. MIRKB contains 7902 entries for 1847 single factors, 195 entries for 157 combined factors and 339 entries for 174 risk models. The single factor types comprise molecular factor (2356 entries, 649 factors), imaging (821 entries, 252 factors), physiological factor (1566 entries, 219 factors), clinical factor (2523 entries, 561 factors), environmental factor (46 entries, 26 factors), lifestyle factor (306 entries, 65 factors) and psychosocial factor (284 entries, 75 factors). Moreover, the database includes five different classification methods of MI, which is more conducive to understanding the risk, development, treatment and prognosis of MI.

Based on our best knowledge, MIRKB is the first online resource to gather all kinds of risk factors and models for MI. In order to build an integrated research platform for studying the interactions of factors, risk models and MI, continued efforts will be made to update the MI data, and to improve the data’s diversity and quality. We will keep the updating of MIRKB and improve and refine the database functionality, and try to build systems level and personalized models for the precision prediction of MI.

## Notes

**Database URL**: http://sysbio.org.cn/MIRKB/
